# Evaluation of the prevalence and risk factors of depression in patients admitted to the CCU of the persian gulf hospital in bandarabbas city

**DOI:** 10.1192/j.eurpsy.2021.912

**Published:** 2021-08-13

**Authors:** N. Seyyedirad

**Affiliations:** Psychiatry, Clinical Research Development Unit (CRDU), Sayd Shirazi Hospital, Golestan University of Medical Sciences, Gorgan, Iran, gorgan, Iran

**Keywords:** Depression, ccu

## Abstract

**Introduction:**

The diagnosis of depression and identifying the factors affecting it in patients with high levels of hospitalization is necessary. Evaluating the prevalence of depression in this population is difficult, because some of the symptoms in depression and medical illness are similar and it is probable the diagnosis of depression to be missed in this patients.

**Objectives:**

The purpose of this study was to investigate the prevalence of depression and factors affecting it in patientsadmitted to the CCU of the Persian Gulf hospital in BandarabbasCity.

**Methods:**

This study was performed on 133 patients admitted to the Persian Gulf Hospital. After obtainingconsent from patients, depression was recorded based on Beck questionnaire (BDI-II). Demographic data was registered from hospital chart review, and patient interview. Chi-Square and Mann-Whitney tests were used to compare the data.

**Results:**

Our results showed that the prevalence of depressionwas significant in CCU patients. 14.3% of CCU patients had moderate to severe depression and 54.59% had mild depression and only 30.8% were normal. Our study also demonstrated that there was a direct and significant relationship between depression and age, low education level, unemployment and length of hospitalization. (P <0.05)

**Conclusions:**

Considering the high prevalence of depression in CCU patients, it is necessary to identify and perform therapeutic measures in patients at high risk for mental illness.

